# Electrochemical Switching of Laser-Induced Graphene/Polymer Composites for Tunable Electronics

**DOI:** 10.3390/polym17020192

**Published:** 2025-01-14

**Authors:** Maxim Fatkullin, Ilia Petrov, Elizaveta Dogadina, Dmitry Kogolev, Alexandr Vorobiev, Pavel Postnikov, Jin-Ju Chen, Rafael Furlan de Oliveira, Olfa Kanoun, Raul D. Rodriguez, Evgeniya Sheremet

**Affiliations:** 1 Research School of Chemical and Biomedical Technologies, Tomsk Polytechnic University, Lenin Ave. 30, 634050 Tomsk, Russia; maksim@tpu.ru (M.F.); ilia.ser.petrov@gmail.com (I.P.); elizavetadogadina@gmail.com (E.D.); kogolev@tpu.ru (D.K.); aov4@tpu.ru (A.V.); postnikov@tpu.ru (P.P.); esheremet@tpu.ru (E.S.); 2 School of Materials and Energy, University of Electronic Science and Technology of China, Chengdu 610054, China; jinjuchen@uestc.edu.cn; 3 Brazilian Nanotechnology National Laboratory (LNNano), Brazilian Center for Research in Energy and Materials (CNPEM), Campinas 13083-970, Brazil; rafael.furlan@lnnano.cnpem.br; 4 Measurement and Sensor Technology, Faculty of Electrical Engineering and Information Technology, Technische Universität Chemnitz, 09126 Chemnitz, Germany

**Keywords:** laser processing, graphene polymer composites, electrochemistry, liquid-gated transistor, tunable properties

## Abstract

Laser reduction of graphene oxide (GO) is a promising approach for achieving flexible, robust, and electrically conductive graphene/polymer composites. Resulting composite materials show significant technological potential for energy storage, sensing, and bioelectronics. However, in the case of insulating polymers, the properties of electrodes show severely limited performance. To overcome these challenges, we report on a post-processing redox treatment that allows the tuning of the electrochemical properties of laser-induced rGO/polymer composite electrodes. We show that the polymer substrate plays a crucial role in the electrochemical modulation of the composites’ properties, such as the electrode impedance, charge transfer resistance, and areal capacitance. The mechanism behind the reversible control of electrochemical properties of the rGO/polymer composites is the cleavage of polymer chains in the vicinity of rGO flakes during redox cycling, which exposes rGO active sites to interact with the electrolyte. Sequential redox cycling improves composite performance, allowing the development of devices such as electrolyte-gated transistors, which are widely used in chemical sensing applications. Our strategy enables the engineering of the electrochemical properties of rGO/polymer composites by post-treatment with dynamic switching, opening up new possibilities for flexible electronics and electrochemical applications having tunable properties.

## 1. Introduction

Graphene and its derivatives have attracted significant attention from researchers during the last two decades [1,2]. Among these derivatives, graphene oxide (GO) is particularly promising in transferring graphene technologies from the lab to real-world applications, primarily due to the possibility of tailoring its physicochemical properties and hydrophilic nature. This hydrophilicity makes GO compatible with different solution-based deposition technologies, such as inkjet printing, spray coating, and screen printing, which are ideal for large-area and batch fabrication [3]. GO can be processed using different routes: chemical, thermal, and photoreduction, which result in reduced graphene oxide (rGO) with partially recovered graphene-like properties [4]. Photoreduction by laser processing offers the benefit of creating well-defined and ad hoc patterning that allows the production of free-form rGO structures with tunable properties.

The unique properties of rGO [5,6], such as tunable electrical conductivity, wettability, and chemical stability, make it attractive as a filler in polymer composites for a wide range of applications, including energy storage, electrocatalysis, bioelectronics, and sensors [7,8,9,10,11,12]. In these applications, the electrochemical properties of rGO play a crucial role. While most research works considering GO and rGO electrochemistry are focused on the applications, several works have also looked at the fundamental electrochemical behavior of GO and rGO [13,14]. However, rGO-based composites have received less attention regardless of their practical relevance, especially for composites obtained by laser processing.

In this work, we investigate the electrochemical properties of laser-induced rGO/polymer composites using polyethylene terephthalate and polyvinylidene fluoride as substrates for GO deposition [15]. We report an unexpected electrochemical behavior observed under potentials exceeding the water electrolysis voltage window. This behavior results in a substrate-predefined “switching” of electrochemical properties such as electrical impedance, charge transfer resistance (R_ct_), and areal capacitance after a few “activation” electrochemical treatment cycles associated with changes in polymer structure. This switching capability, arising from the electrolyte interaction with freshly exposed rGO, provides a new degree of freedom for tailoring the electrochemical properties of rGO/polymer composites beyond what is achievable by sole laser processing since laser processing, in particular for composite formation has only a small window of parameters to achieve the composite while preserving the structural integrity of both components. In other words, the ability to control properties is limited by the composite formation mechanism [15]. Moreover, after the “activation” cycles, the rGO/polymer composite properties can be reversibly changed upon consecutive electrochemical treatment. This electrochemical treatment allows the development of an electrolyte-gated transistor that otherwise did not work when using non-treated electrodes. The mechanism behind this switching behavior is related to electrochemical polymer erosion, which exposes rGO to the interaction with the electrolyte.

## 2. Materials and Methods

### 2.1. Materials

Graphene oxide water dispersion with a concentration of 4 mg mL^−1^ (monolayer content >95%, measured in 0.05 wt%; particle size: D90 5–7 µm, D50 2–4 µm, D10 1–2 µm; Graphenea, San Sebastián, Spain); phosphate-buffered saline (PBS) with pH of 6.86 (Uralkhiminvest, Ufa, Russia); PET sheets with a thickness of 0.65 mm were purchased in local store; PVDF was prepared using fused deposition modeling 3D printing.

### 2.2. Laser-Induced Composite Fabrication

Both rGO/PET and rGO/PVDF were fabricated following the same protocol. First, GO was drop-casted on a polymer substrate at 90 µL cm^−2^ and left for drying on a heating plate under 40 °C in air. The dry films with a 30 µm GO thickness (Figure S1) were laser processed using a pulsed diode laser with a wavelength of 436 nm operating at 1.6 kHz frequency (pulse energy ~0.17 J, pulse width ~260 µs). After laser processing, the composite structures were sonicated in distilled water for 5 min to remove GO residues and poorly integrated rGO.

### 2.3. Electrochemical Treatment and Characterization

All the electrochemical experiments were conducted in the three-electrode cell with Pt wire, Ag/AgCl (saturated KCl), and rGO/polymer composite as the counter, reference, and working electrodes. PBS (pH 6.86) was used as an electrolyte throughout all the experiments. Data were recorded using Corrtest CS2350M (Corrtest, Wuhan, China) bipotentiostat-bigalvanostat with the impedance measuring module. Electrochemical impedance spectroscopy (EIS) measurements were carried out with a sinusoidal waveform with a 0 V DC component and a 50 mV AC amplitude. This higher-than-typical amplitude (10–20 mV) was necessary to ensure a sufficient signal-to-noise ratio, as the electrodes, especially after electrochemical treatment, showed resistances of several tens of kOhm). The EIS measurements were performed within the 100 kHz–0.25 Hz frequency range. R_ct_ was determined as an intercept of a semicircle range with the Z’ axis in the Nyquist plot. Cyclic voltammograms (CVs) were measured with a scan rate of 100 mV s^−1^. Potential windows are specified in the manuscript text. Areal capacitance was calculated from the CVs using the following equation:

(1) $$C=\frac{\int idV}{2\cdot \Delta E\cdot A\cdot W}$$

where ∫idV is the integral over CV, Δ*E* is a potential window, *A* is the geometrical area of the electrode, and *W* is a scan rate.

### 2.4. Electrolyte-Gated Transistor (EGT) Demonstration

The EGT performance was demonstrated by employing a Zurich MLFI Lock-in Amplifier (Zurich Instruments, Zurich, Switzerland). The EGT channel dimensions were approximately 2 × 2 mm^2^. Drain and source contacts were made with copper tape and conductive silver paste and encapsulated with polydimethylsiloxane (PDMS). The applied drain-source voltage (V_DS_) was −0.5 V, and the gate-source voltage (V_G_) sweep rate was 25 mV s^−1^. Transfer characteristics were recorded in the V_G_ range spanning from −0.6 to +0.6 V. Gate voltage equals +0.5 V was used for sensing KCl with different concentrations. This value provides the maximum sensitivity and evades the electrolysis and reduction processes. Pt wire was used as a gate electrode.

EGT operation was performed following the steps: First, a 20 μL deionized (DI) water drop was deposited on the EGT channel, and the I_D_ current was recorded for 1 min. The liquid was then removed with absorbing paper, and a 20 μL drop of 50 mM KCl was added in the same way. The procedure was repeated for 100 mM and 200 mM KCl solutions.

### 2.5. Scanning Electron Microscopy

The scanning electron microscopy (SEM) was performed using the Tescan Mira 3LMU (Tescan, Brno, Czech Republic) and COXEM EM-30+ (COXEM, Daejeon, Republic of Korea). All the SEM images shown in the manuscript were acquired by detecting the secondary electrons. The top-view SEM images were acquired with 10^3^× magnification, while cross-section images for rGO/PET and rGO/PVDF were acquired with 200× and 190× magnification, respectively.

### 2.6. X-Ray Photoelectron Spectroscopy

The X-ray photoelectron spectroscopy (XPS) was performed using a Thermo Fisher Scientific XPS NEXSA (Thermo Fisher Scientific, Waltham, MA, USA) spectrometer with a monochromatic Al K Alpha X-ray source at 1486.6 eV. The survey study was carried out employing a pass energy of 200 eV and an energy resolution of 1 eV. For the high-resolution spectra, the pass energy was 50 eV, and the energy resolution was 0.1 eV with a spot diameter of 400 μm.

### 2.7. Raman Spectroscopy

Raman spectra were recorded using a confocal Raman microscope (NTEGRA Spectra, NT-MDT, Zelenograd, Russia) with 532 nm (for rGO/PVDF) and 633 nm (for rGO/PET) diode excitation lasers. The Mitutoyo objective with 20× magnification was used to focus a laser beam on the sample surface and record spectra with 1 s exposure time and 10 times accumulation.

### 2.8. Fourier-Transform Infrared Spectroscopy

FTIR was measured on the IRAffinity-1S (Shimadzu, Kyoto, Japan) spectrometer in the total internal reflection mode.

## 3. Results

### 3.1. Electrode Fabrication and Characterization

**Figure 1:**
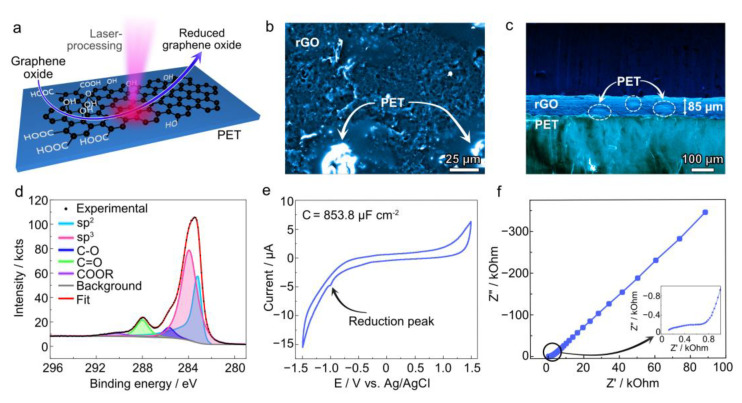
(**a**) Schematic illustration of the electrode fabrication procedure. (**b**) SEM top-view image of rGO/PET composite electrode. (**c**) Cross-sectional SEM image of rGO/PET composite electrode. (**d**) C1s high-resolution XPS spectrum from rGO/PET composite electrode. (**e**,**f**) CV and Nyquist plots, respectively, measured using the rGO/PET composite electrode. Inset represents the zoom into the high-frequency region where semicircle charge transfer resistance is observed.

rGO/PET composite electrodes were fabricated by laser processing drop-casted GO films on a PET substrate (Figure 1a). The fabrication protocol and the mechanism behind the composite formation are presented in detail elsewhere [9,15]. Briefly, GO acts as a photothermal transducer during laser irradiation, partially converting light into thermal energy. This energy reduces GO into rGO and partially becomes transferred to the polymer substrate underneath, reducing its viscosity and resulting in rGO intermixing with the liquified polymer due to the Benard–Marangoni convection. This process generates a conductive carbon network encapsulated by the polymer surface.

SEM imaging reveals two different regions after laser processing, in agreement with our previous work [9]: (i) a rough, porous region with low static charging attributed to conductive rGO and (ii) a brighter region with strong static charging attributed to PET islands (Figure 1b). This surface structure has been confirmed by optical microscopy, which also shows the encapsulated black carbon material visible through the transparent PET layer (Figure S2). Cross-sectional SEM imaging also reveals an rGO/PET composite film with a thickness of ca. 80 µm lying on the PET substrate, with some PET islands visible as bright spots within the composite layer (Figure 1c).

The survey XPS spectrum of the rGO/PET electrode (Figure S3) shows the characteristic C1s and O1s bands, with a small amount of sulfur—a residual byproduct from the GO synthesis. High-resolution C1s spectra (Figure 1d) were deconvoluted into peaks related to sp^2^ carbon (283.2 eV), sp^3^ carbon (283.9 eV), C-O (285.7 eV), C=O (288.0 eV), and -COOR (290.3 eV). Below, we discuss the relative content of these elements and functional groups.

The electrochemical properties of the electrodes have been characterized by cyclic voltammetry and electrochemical impedance spectroscopy. CV (Figure 1e) exhibits nearly pure capacitive behavior with a small reduction peak at approximately −1 V (vs. Ag/AgCl), likely due to the reduction of aldehyde moieties [13] and signs of water electrolysis. Areal capacitance (C_A_) has been set as the figure of merit to compare the induced changes. The as-prepared electrode exhibited a C_A_ of 1.15 ± 0.74 mF cm^−2^, slightly lower than the state of the art for rGO [16], possibly due to the partial coverage of rGO active sites by dielectric PET islands. The R_ct_ and impedance at 0.25 Hz (|Z|_0.25_) extracted from the Nyquist plot have been used as figures of merit for EIS. R_ct_ calculated from the Nyquist plot (Figure 1f) was several kΩ, followed by a linear region characteristic of diffusion through a semi-infinite diffusion layer, resulting in high impedance values at low frequencies (Figure 1f).

### 3.2. Unexpected Findings on the Electrochemical Behavior of rGO/PET Electrodes

**Figure 2:**
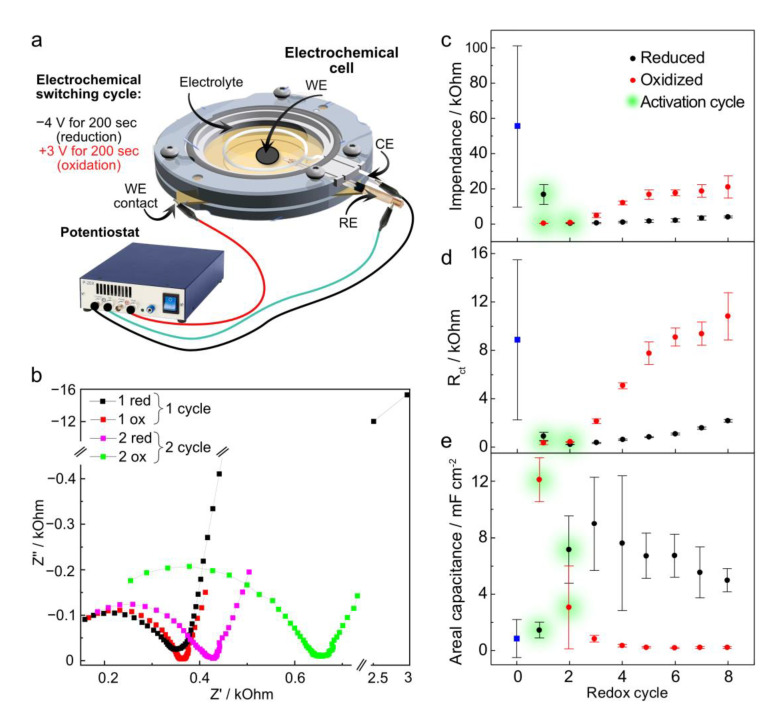
(**a**) Schematic illustration of the experimental setup representing the electrochemical cell and a short description of a sequential electrochemical treatment. (**b**) Nyquist plots from rGO/PET samples after the first two redox cycles, indicating the observed changes in impedance. (**c**–**e**) rGO/PET sample figures of merit were obtained from CVs and impedance plots through all eight investigated redox cycles: impedance value at 0.25 Hz, charge transfer resistance, and areal capacitance, respectively. The values at the “0” point represent the values for the initial samples, which are also marked with blue squares. The error bars show the STD of the three electrodes.

To explore the electrochemical properties of the rGO/PET electrodes, we carried out reduction and oxidation cycles on the sample using a three-electrode cell. We applied high constant voltages of different polarities (−4 V for reduction and +3 V for oxidation) for 200 s each (Figure 2a) and measured CVs and the impedance after each treatment step. We performed eight full treatment cycles, each including one reduction and one oxidation step. The first two cycles, referred to as “activation” cycles, exhibited distinctive behavior in CVs, current versus time curves (i–t), and Nyquist plots (Figure 2b). Subsequent cycles are denoted as “switching” due to the changes observed in |Z| at 0.25 Hz, R_ct_, and areal capacitance (Figure 2c–e). The values obtained from these experiments are summarized in Table S1.

#### 3.2.1. Activation Cycles

During the first redox cycles, we observed a significant drop in both |Z|_0.25_ and R_ct_ from kΩ (initially in the range of several kΩ to dozens of kΩ) to several hundred Ω upon applying both positive and negative potentials (Figure 2b). At the second redox cycle, both |Z|_0.25_ and R_ct_ start to increase slightly. Interestingly, the sample areal capacitance depends on the polarity of the applied potential, rising by a factor of ~6 within the first two reduction cycles and further decreasing by one order of magnitude after the oxidation step. Importantly, these changes occurred only if the reduction step was performed first. Otherwise, starting with the oxidation step resulted in electrode degradation, as shown by the CV in Figure S4, limiting the possibility of obtaining meaningful data.

First red: CVs from the initial samples exhibit mostly capacitive behavior, with a weak reduction peak found at ca. −1 V, a signature of the electrochemical reduction of residual carbonyl and carboxyl groups remaining after laser modification. Applying the reduction potential (−4 V) results in a typical hydrogen evolution reaction i–t curve, with the current decaying over time (Figure S5, 1st red). After the first reduction step, the electrodes still exhibit pure capacitive behavior, but with no evident peaks in the CVs and with an increase in capacitance contribution (Figure S6, 1st red; Figure 2e).

First ox: The oxidation step with +3 V shows signs of a concomitant oxygen evolution reaction, as indicated by the slight current increase during the first 50 s followed by a decay up to 200 s (Figure S5, 1st ox). The CV recorded after the first oxidation step shows a further increase in the capacitive contribution and the appearance of symmetrical oxidation and reduction peaks at ±0.5 V.

Second red: The i–t curve during the second reduction exhibited behavior similar to that of the 1st red, which is related to the water-splitting process. Even though the underlying processes are similar, they occur differently since the 2nd red i–t curve shows a steep current decay, contrary to the 1st red, associated with an enhanced diffusion of water molecules toward the electroactive rGO network. However, CV recorded after the second reduction reveals the same oxidation and reduction peak profile at the same voltages as observed after the first oxidation step.

Second ox: The second oxidation process differs from previous modifications and shows two distinguishable current peaks in the i–t curves during the first 100 s, indicative of electrochemical reactions. These reactions shift the equilibrium cathodic potential toward zero, as evidenced by the intercept of the backward and forward scan curves in the CV at ~−0.8 V, as well as dynamic changes in CVs from cycle to cycle (Figure S7). To ensure that CV measurements did not significantly affect the changes introduced by the oxidation treatment, we narrowed down the potential range after oxidation from ±1.5 V to ±0.5 V. This range was selected because most available literature, including ours, indicate no redox processes within this range [17]. The CV measuring range for reduction cycles was not changed since keeping the same range did not affect the figures of merit. After narrowing the potential range, we observed a strong reduction peak on the i–t curve during the fourth reduction step, signaling a strong ongoing electrochemical reduction, most likely coming from processes in rGO (Figure S5). This peak confirms the necessity of narrowing the CV potential window after oxidative treatment.

We performed a set of in-depth characterization to understand the structural changes during the “activation” period. Since we did not observe reaction signatures in the i–t curves, we suspected morphological changes, such as electrochemical roughening, could explain the electrode behavior. SEM images show that electrochemical treatment significantly decreases the number of PET fragments, as indicated by the contrast between treated and untreated regions of the sample, while the initial sample had PET islands homogeneously distributed all over the surface (Figure S8). This result is a sign of electrochemical destruction of the PET. We calculated the ratio between sp^2^ and sp^3^ hybridized carbon atoms from XPS C1s spectra and compared it to the untreated sample with a sp^2^/sp^3^ ratio of 0.88 ± 0.71.

XPS spectra from the sample after the first reduction step show the typical rGO peaks. The C1s high-resolution region (Figure S9) was deconvoluted into bands corresponding to sp^2^ and sp^3^ hybridized carbon (283.4 and 284.3 eV, respectively), C-O (285.7 eV), C=O (288.3 eV), -COOR (290 eV), and the π-π* satellite (292 eV). The calculated sp^2^/sp^3^ ratio is 1.67 ± 0. 77, almost double that of the initial sample (Figure 1d). This increase correlated with an increase in the C/O ratio from 4.42 ± 0.77 (initial) to 5.09 ± 0.31 (1st red). The survey spectrum also revealed the presence of sodium in the sample, likely originating from sodium cations appearing on the surface of PET due to cleavage of ester bond and formation of -COONa groups (PBS electrolyte contains NaCl). The following first oxidation step shows ion trapping from the electrolyte, evidenced by the appearance of phosphorus (0.66 ± 0.08 atomic %) and chlorine (1.00 ± 0.02 atomic %) in the survey spectra. Additionally, the sodium content increased slightly due to entrapment of sodium phosphate and chloride. The most remarkable result from this oxidation step is that the sp^2^/sp^3^ carbon ratio remained unchanged, while the C/O ratio drastically decreased to 3.38 ± 0.07. This is surprising because an increase in oxygen content is typically related to the change in carbon hybridization from sp^2^ to sp^3^.

The second reduction and oxidation cycles did not change the sample elemental composition but induced changes in their states. The second reduction significantly increases the sp^2^/sp^3^ ratio to 5.85 ± 0.71 (by a factor of 3.3) while only slightly affecting the C/O ratio (increased from 3.38 ± 0.07 to 3.54 ± 0.20). This demonstrates that the chemical reactions occurred within the electrode’s structure. Other elements’ content from the electrolyte (Cl^−^, PO4^−^, Na^+^) changed according to the polarity of the applied potential, as summarized in Table S2. The second oxidation decreased the sp^2^/sp^3^ ratio to 1.45 ± 0.02, while the C/O (3.57 ± 0.05) remained unchanged.

These results are surprising because changes in the sp^2^/sp^3^ atomic ratio in these systems are usually explained by the formation or cleavage of chemical bonds (reflected by a decrease or increase in sp^2^/sp^3^, respectively) between carbon and oxygen-containing groups. This demonstrates that the underlying mechanism differs from the typical reduction and oxidation of GO reported in the literature.

#### 3.2.2. Switching Cycles

Further electrochemical treatment cycles result in reversible “switching” of the electrochemical properties. Applying the reduction potential led to |Z|_0.25_ values of ~3 kΩ. In contrast, oxidation potentials caused a constant increase in |Z|_0.25_ up to the 5th cycle, after which the impedance stabilized around 20 kΩ for all following oxidation treatments (Figure 2c). A similar trend is observed for R_ct,_ with the reduced state stabilizing around 1 kΩ and the oxidized state stabilizing at the 5th cycle with values close to 10 kΩ (Figure 2d). For the areal capacitance, the oxidized state values were 0.5 mF cm^−2^. In contrast, the reduced state exhibited a one-order-of-magnitude increase (Figure 2e).

Analysis of the impedance curves revealed that, after the first two activation cycles, applying the reduction potential leads to the appearance of a second semicircle in the 3rd red Nyquist plot (Figure S10a) with a maximum (~15°) in the phase angle plot located around 1 kHz. The phase angle at frequencies below 1 Hz does not exceed 15° (Figure S10b). The second semicircle (representing an imperfect double-layer capacitor) at the mentioned position with a low phase angle value could be a signature of rGO, as previously observed for photoreduced GO [14]. After oxidation treatment, the second semicircle shifts from 1 kHz toward lower frequencies, with the phase angle rising up to 30°, indicating oxidation of the reduced graphene oxide (Figure S10b).

To determine the mechanism behind this “switching” of electrochemical properties, we characterized the samples up to the 5th oxidation since the key values for all states stabilized at this cycle. We first recorded Raman spectra (Figure S11) showing that the exposed regions (not covered with PET) were characterized by D and G peaks, defect-activated band around 1350 cm^−1^, and sp^2^ carbon band at 1590 cm^−1^, respectively. The intensity ratio between these bands (I_D_/I_G_) has often been used to estimate the defect concentration in graphenes. For GO and rGO, this ratio is related to the degree of reduction since oxygen-containing groups are structural defects [18].

The initial rGO/PET electrode shows an uneven distribution of Raman spectra at different locations, with some regions showing high-quality rGO with I_D_/I_G_ much lower than 1, while others had higher I_D_/I_G_ ratios due to highly defective rGO. After the first reduction, the I_D_/I_G_ was calculated to be 0.77, which dropped to 0.57 after the first oxidation, following the trend observed in the electrochemical properties where impedance decreased with both the first reduction and oxidation. Surprisingly, the second reduction resulted in a significant increase in I_D_/I_G_ to 1.82, while further electrochemical treatment resulted in only minor fluctuations in I_D_/I_G_ within the error margin. It is important to note that I_D_/I_G_ can be insensitive to changes in chemical composition for highly defective rGO structures [19,20], which might be the case here since electrochemical treatment could result in a high number of defects. Therefore, we needed more than the Raman spectra to fully elucidate the underlying switching mechanism.

Considering the relatively high potentials and the nature of the PET substrate, we hypothesized that the unusual electrochemical behavior accompanied by changes in the sp^2^/sp^3^ ratio but not the C/O ratio could be attributed to PET’s electrochemical activity; the particular mechanism is discussed below.

We replaced PET with the electrochemically inert polymer polyvinylidene fluoride (PVDF) to elucidate the individual contributions of rGO and PET to this unusual electrochemical behavior. This comparison was possible due to the similar mechanism behind the laser-induced rGO composite formation with PET and PVDF [15].

### 3.3. Electrochemical Treatment of rGO/PVDF Electrodes

To determine whether the observed electrochemical switching originates from rGO alone or involves some kind of interaction between the rGO surface and specific functional groups in polymer chains, we fabricated rGO/PVDF electrodes using the same laser-induced reduction method, as well as sequential electrochemical reduction and oxidation. Before analyzing the electrochemical properties, we investigated the morphology and chemical composition of the rGO/PVDF composite.

**Figure 3:**
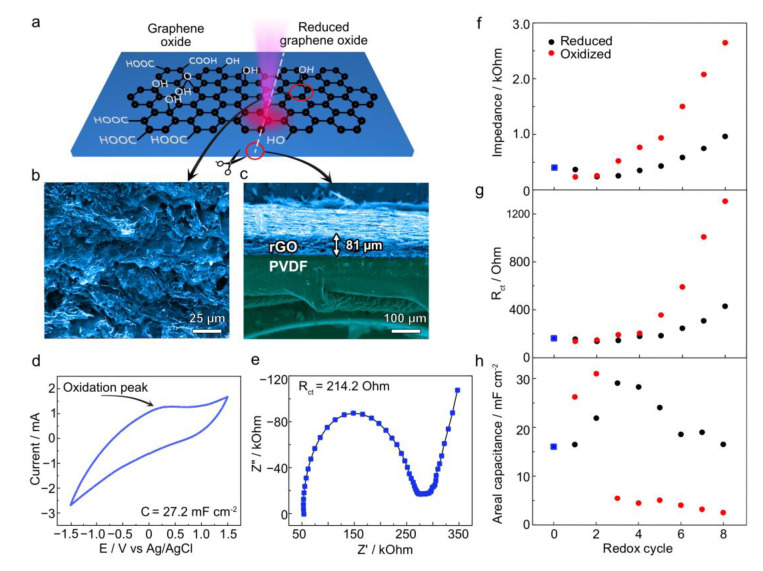
(**a**) Schematic illustration of the rGO/PVDF preparation via laser treatment with the sample (**b**) top-view and (**c**) cross-section obtained by SEM. (**d**,**e**) CV and Nyquist plot for the initial untreated sample, respectively. (**f**–**h**) Plots of calculated sample figures of merit were obtained from CVs and impedance plots through all the investigated 8 redox cycles: impedance values at 0.25 Hz, charge transfer resistance, and areal capacitance, respectively. The values at the “0” point represent the values for the initial samples, which are also marked with blue squares.

SEM analysis shows a distinct morphology for rGO/PVDF compared to rGO/PET. The rGO/PVDF composite is represented by rGO flakes without noticeable polymer presence on the electrode surface (Figure 3b). Cross-sectional SEM images (Figure 3c) reveal two clearly separated regions: a PVDF layer and a highly porous rGO layer with a thickness of ca. 81 µm on top. This implies that the electrochemical behavior of rGO/PVDF (Figure 3d,e) is dominated by the exposed rGO since there are no visible signs of rGO encapsulation by PVDF, and PVDF is an electrochemically inert material.

We performed an XPS analysis to further investigate the presence of polymer on the electrode’s surface. In contrast to rGO/PET, fluorine in PVDF allows for the differentiation between rGO and PVDF in XPS data easily. The survey XPS spectra reveal F1s, C1s, and O1s peaks at 687.5, 285.1, and 532.9 eV (Figure S12). High-resolution C1s spectra were deconvoluted into seven peaks attributed to sp^2^ and sp^3^ hybridized carbon, C-O, C=O, -COOR, CF_2_, and CF_3_ at 284.3, 285.2, 286.9, 287.5, 288.5, 290.4, and 292.8 eV (Figure S12). The presence of a peak corresponding to fluorine in the XPS spectra could be explained through the sorption of low molecular weight PVDF destruction products formed as a result of intense heating of the PVDF substrate during the laser reduction of GO [21].

These differences in surface structure resulted in a significantly lower impedance (ca. 300 Ω) of rGO/PVDF compared to the rGO/PET composite. This is attributed to a smaller Warburg impedance related to a higher ion diffusion on the exposed rGO flakes in the PVDF composite (Figure 3d). The R_ct_ is also approximately one order of magnitude lower. Furthermore, the areal capacitance exhibits values one order of magnitude higher than that of rGO/PET, consistent with the greater exposure of the electroactive rGO network to the electrolyte.

The first two redox cycles did not significantly change the electrochemical parameters set as the figures of merit (Figure 3f–h). However, further treatment shows a similar trend to rGO/PET, although much less pronounced. For instance, impedance varied in rGO/PVDF from ~1 kΩ to ~3 kΩ, compared to switching from ~3 kΩ to ~20 kΩ for rGO/PET. Contrary to rGO/PET showing stable reduced and oxidized states after five cycles, the parameters extracted from EIS for rGO/PVDF diverged with each cycle without reaching stable values.

The initial CV of rGO/PVDF shows a prominent oxidation peak around 0–0.2 V (Figure 3d). The phase angle plot exhibits a well-pronounced extremum (−32°) in the high-frequency region related to charge transfer resistance and a rise in phase up to −23° at low frequencies, indicating residual oxygen functionalities in the graphene structure remaining after laser processing [14]. The first reduction did not cause significant changes in the CV or impedance plots. However, the first oxidation increased the areal capacitance and changed the CV shape to a predominantly capacitive behavior with slight reduction and oxidation peaks between ±0.5–1 V (Figure S13). The impedance decreased by 100 Ω with the phase at a low frequency not exceeding −10°, possibly due to the electrochemical roughening of the electrode’s surface, which increased the exposure of highly reduced conductive active sites. The second redox cycle does not cause any changes in the impedance plots or CVs.

We narrowed the potential window from the third cycle for measuring CV as performed for rGO/PET. We observed clear signs of rGO reduction and oxidation, with low-frequency phase values reaching −30° after oxidation and −12° after reduction. All CVs, i–t curves, and impedance data are presented in Figures S13–S15.

SEM images from samples subjected to several redox cycles revealed increased surface roughness (Figure S16), which might be responsible for the increased capacitance. Although visually treated and untreated regions might not be well distinguished, the extracted surface profiles confirm the significant difference in roughness introduced by electrochemical treatment. We performed XPS analyses following the same procedure as for rGO/PET to investigate the chemical changes. We calculated the C/O ratio as the figure of merit (Table S3), surprisingly showing that the initial sample (ca. 15.2 ± 0.5) had a C/O ratio several times higher than that for rGO/PET and most reports in the literature for rGO obtained using similar laser wavelengths [22]. This demonstrates that the C/O is not a reliable indicator of rGO structure when laser-reduced on a PET substrate, as the oxygen of PET itself can significantly influence it.

In rGO/PVDF, the first reduction slightly decreased the C/O ratio to 9.0 ± 0.1 (Table S3), possibly due to removing poorly integrated but highly reduced rGO flakes from the electrode surface. The first oxidation further decreases the C/O ratio to 5.7 ± 0.3. Further electrochemical treatment resulted in the expected behavior: reduction potentials increase the C/O ratio, while oxidation decreases it, consistent with the electrochemical reduction and oxidation of rGO absent of underlying PVDF substrate.

### 3.4. Mechanism Behind Electrochemical Properties Switching

We propose a mechanism for switching electrochemical properties based on the observed differences between rGO/PET and rGO/PVDF. The rGO/PVDF served as a control system to isolate the contribution of rGO. Over eight redox cycles, rGO/PVDF could be reversibly reduced and oxidized, consistent with previous results on GO obtained using the modified Hummers method [23,24]. This was reflected in the impedance plots, particularly in the phase angle behavior and the CVs, where weak, symmetrical reduction, and oxidation peaks replaced the initial oxidation peak. Although these redox reactions resulted in “switching”, the effect was much less pronounced than in rGO/PET.

We hypothesize that the switching mechanism is related to PET hydrolytic decomposition. Cathodic decomposition of PET occurs through a two-electron reaction with peaks at −2.5 and −3 V [25]. The switching effect appeared only at potentials exceeding ±2.5 V, further supporting PET’s participation in the electrochemical reactions since rGO undergoes redox reactions at lower potentials. Thus, the depolymerization begins during the first redox cycle (activation), and there is no switching effect. As the destruction of thin polymer layers progresses, more conductive rGO is exposed to the electrolyte, increasing the areal capacitance and decreasing impedance, mainly by reducing the diffusional component. Further cycles started showing the switching behavior due to rGO redox reactions of the freshly exposed flakes. However, we do not exclude the possibility of a cascade of various processes involving PET and rGO, such as hydrolysis, inter-material diffusion, and reactions of oxygen-containing groups.

The difference in behavior between rGO/PET and rGO/PVDF could be explained by the presence of polymer on the composite surface. In rGO/PET, erosion exposes a limited amount of the conductive rGO network, significantly contributing to the electrode/electrolyte interface. In contrast, the rGO/PVDF surface is dominated by rGO, where oxidation is a more statistically distributed process, affecting only some regions on the surface and leading to less pronounced changes at the electrode/electrolyte interface. This, along with the electrochemical inertness of PVDF and limited electrolyte diffusion through the rGO layer, contributes to the hindered switching behavior observed in the rGO/PVDF composite.

### 3.5. Application of Laser-Processed rGO/Polymer Composites in Electrolyte-Gated Transistors

rGO is widely used in chemical sensors and biosensors, where its electrochemical properties define the device’s performance. However, the use of rGO/polymer composite is limited since the polymer matrix can significantly hinder the electrode/electrolyte interactions, preventing exposure to active sites. We exploit the electrochemical “activation” phenomenon discovered here to expose rGO in an rGO/PET composite for application as an EGT [26]. EGTs are formidable electronic transducers widely employed as chemical sensors, biosensors, and neuromorphic devices for bioelectronics [27]. They bear very interesting characteristics such as low voltage operation (<1 V), elevated sensitivity to surface perturbations, and signal amplification ability [28]. The EGT working principle relies on the modulation of the charge carriers at the transistor channel via the formation of electrical double layers at the device interfaces (field-effect) or ion intake (electrochemical mode), which are controlled by the gate voltage (V_G_) [28,29,30,31].

To demonstrate the impact of electrochemical treatment on rGO/polymer composites, we compared the performance of initial rGO/PET and electrochemically-treated (1st red, 1st ox, 3rd red, and 3rd ox cycles) electrodes as a channel material in EGTs. Our strategy enabled the fabrication of millimeter-scale EGTs with rGO channels and adjustable electronic properties. The best performance was obtained for the 1st red sample, which we discuss further below.

**Figure 4:**
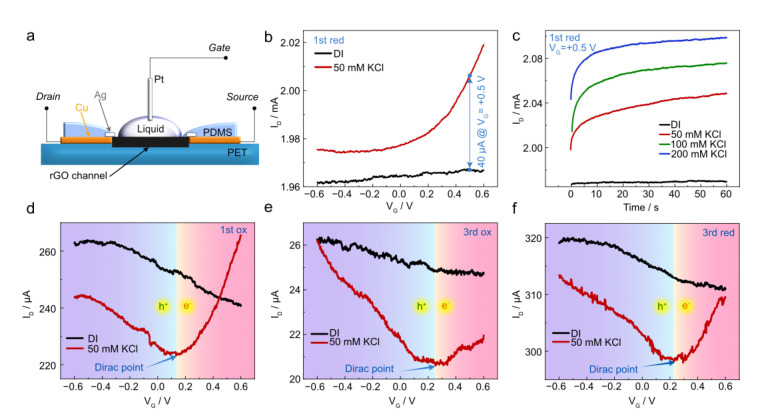
(**a**) Schematic illustration of the setup for transistor characteristics measurements. (**b**) Transfer characteristics in DI water and 50 mM KCl liquid gate for 1st red sample at V_DS_ = −0.5 V. (**c**) Device response at V_G_ = +0.5 V and V_DS_ = −0.5 V corresponds to different KCl molarity for 1st red samples. (**d**–**f**) Transfer characteristics in DI water and 50 mM KCl for 1st ox, 3rd ox, and 3rd red samples, respectively.

The EGT schematic representation is shown in Figure 4a. We first recorded the device transfer characteristics, i.e., the drain current (I_D_) vs. V_G_ in deionized water (DI) and 50 mM KCl solution. The initial rGO/PET sample showed no current modulation under ±0.6 V V_G_ bias voltage either in DI or KCl (Figure S17a). This result is attributed to the polymer shielding the rGO surface, suppressing the formation of an electrical double-layer or ion intake within the sample. In contrast, the 1st red sample showed significant I_D_ modulation, especially at positive V_G_. For example, at a positive bias of V_G_ = 0.5 V, a 40 μA difference in I_D_ is observed between operation in DI water and in 50 mM KCl solution (Figure 4b). This current modulation shows that PET erosion during the 1st red step activates the surface, allowing the interaction between electrolyte ions and the rGO channel.

To further demonstrate the robustness of EGTs produced from the electrochemically activated composite, we measured I_D_ over time at different KCl concentrations. The initial rGO/PET sample showed no sensitivity to the electrolyte composition (Figure S16b). The 1st red sample showed a reasonably stable response over time at different KCl concentrations (50, 100, and 200 mM) as depicted in Figure 4c. Increasing the electrolyte concentration increases the electrical double-layer capacitance and, therefore, I_D_. A stable current response is a key characteristic in sensing, especially for applications targeting real-time monitoring of chemical species. Transfer characteristics of the 1st ox, 3rd red, and 3rd ox samples are presented in Figure 4d–f. We observe a clear charge neutrality point near +0.2 V and the typical rGO ambipolar electrical conduction here, illustrated by the p- and n-type branches of the transfer curve. From the device transfer characteristics, we calculated the hole (µ_h_) and electron mobility (µ_e_) in EGTs based on rGO/PET composites (Note S1). We found µ_h_ to amount to 0, 5.9⋅10^−3^, 5.6⋅10^−3^, and 1.6⋅10^−2^ cm^2^ V^−1^ s^−1^, and µ_e_ was found equal to 0.15, 1.6⋅10^−2^, 0.9⋅10^−2^, and 0.8⋅10^−2^ cm^2^ V^−1^ s^−1^ for the 1st red, 1st ox, 3rd red, and 3rd ox, respectively. Details on the calculation of the charge carrier mobilities are given in the Supporting Information. The µ_e_ for the 1st red sample showed state-of-the-art values for rGO [32], although p-type conduction could not be obtained, possibly due to hole trapping at the interface. Our method demonstrates the potential of electrochemically-activated laser-reduced GO/polymer composites to be used in the fabrication of EGT with potential for sensing applications.

## 4. Conclusions

In this work, we investigated the electrochemical behavior of laser-induced rGO/PET composite electrodes under harsh electrochemical conditions, using potentials exceeding the water window. We found that this treatment gives rise to unexpected changes in the composite electrode’s structure, enabling the tuning of electrochemical properties, including impedance, charge transfer resistance, and areal capacitance. This effect originates from the unique composite structure, with a high surface coverage of PET, due to the Marangoni effect during laser processing, which initially hinders the availability of rGO active sites. The electrochemical treatment involves two processes. First is an activation period where PET erosion and depolymerization expose rGO to the electrolyte. Second, a switching period in which freshly exposed rGO undergoes reversible redox reactions, as deduced from the behavior of rGO/PVDF composite electrodes, where rGO dominates the electroactive surface. The difference in switching efficiency between rGO/PET and rGO/PVDF is attributed to the electrochemical activity of PET and its role in interrupting the conductive rGO network, enhancing the impact of electrochemical treatment on the overall electrode properties.

This electrochemical treatment method, coupled with laser-induced GO reduction and free-form fabrication, provides a new approach to engineering the electrochemical properties of rGO/polymer composites. We illustrated the ability to control ion transfer through PET erosion, opening possibilities for applications such as electrolyte-gated transistors for ion sensing.

## Data Availability

The original contributions presented in the study are included in the article/Supplementary Material, further inquiries can be directed to the corresponding author.

## Supplementary Materials

The following supporting information can be downloaded at https://www.mdpi.com/article/10.3390/polym17020192/s1, Figure S1. Cross-section optical microscopy image of the GO/PET (sample before laser treatment). Figure S2: Optical microscopy image of pristine rGO/PET electrode; Figure S3. XPS survey spectrum from the rGO/PET pristine sample; Table S1: Calculated values from CVs and Impedance plots after electrochemical treatment; Figure S4: Typical CV after the first reduction in a following sequence 1st ox-1st red (treatment was started with the oxidation step); Figure S5: i–t curves from the rGO/PET electrode during each of the first 5 redox cycles; Figure S6: CVs from rGO/PET electrode after each of the first 5 redox cycles; Figure S7: CV after the third oxidation cycle without changing the potential window; Figure S8: SEM images of the initial sample and sample after the 2nd oxidation. The red mask was created as a grain analysis to illustrate PET islands. Dashed squares show regions from which the profiles were extracted, while the white circle represents the electrochemically treated region; Figure S9: Survey and C1s XPS high-resolution spectra from rGO/PET electrode after two redox cycles. The tables show values of relative atomic content averaged from 3 different points per sample; Table S2: Atomic percent of elements from XPS Survey spectra for rGO/PET samples after first two redox cycles; Figure S10: (a) Nyquist plots and (b) phase angle plots from the rGO/PET electrode after each of the first 5 redox cycles; Figure S11: Raman spectra from the rGO/PET electrodes after each of 5 electrochemically treatment cycles; Table S3: C/O ratio extracted from XPS survey spectra from different treatment cycles of rGO/PVDF; Figure S12: Survey and C1s XPS high-resolution spectra from rGO/PVDF electrode after several redox cycles; Figure S13: CVs from rGO/PVDF electrode after each of the first 5 redox cycles; Figure S14: i–t curves from rGO/PVDF electrode after each of the first 5 redox cycles; Figure S15: (a) Nyquist plots and (b) phase angle plots from the rGO/PVDF electrode after each of the first 5 redox cycles; Figure S16: SEM of rGO/PET electrode after several redox cycles; Figure S17: (a) Transfer characteristic of pristine rGO/PET in DI and 50 mM KCl. (b) Respective current-time at V_G_ = +0.5 V and V_DS_ = −0.5 V for varied KCl concentrations; Note S1: Charge carriers’ mobility for all the samples was calculated according to the Formula (S1), $\mu =\frac{\Delta I_{D}}{\Delta I_{G}}\cdot \frac{L}{W\cdot V_{DS}\cdot C}$ (S1), where μ—charge carriers mobility, $\frac{\Delta I_{D}}{\Delta I_{G}}$—slope of the linear region of transfer characteristic, L, W—the length and the width of a channel, V_DS_—source-drain voltage, and C—specific capacitance.
