# Supplementary material for: Electrochemical Switching of Laser-Induced Graphene/Polymer Composites for Tunable Electronics
Source: Polymers (Basel). 2025 Jan 14;17(2):192. doi: 10.3390/polym17020192 (PMC11768407; doi:10.3390/polym17020192)
Supplement: Supplementary file 1 [file polymers-17-00192-s001.zip › polymers-3331857-supplementary.pdf]

polymers

Supporting Information

Electrochemical Switching of Laser-Induced Graphene/Polymer Composites for Tunable Electronics

Maxim Fatkullin [1], Ilia Petrov [1], Elizaveta Dogadina [1], Dmitry Kogolev [1], Alexandr Vorobiev [1], Pavel Postnikov [1], Jin-Ju Chen [2], Rafael Furlan de Oliveira [3], Olfa Kanoun [4,*], Raul D. Rodriguez [1,*] and Evgeniya Sheremet [1]

1 Research School of Chemical and Biomedical Technologies, Tomsk Polytechnic University, Lenin Ave. 30, 634050 Tomsk, Russia; maksim@tpu.ru (M.F.); ilia.ser.petrov@gmail.com (I.P.); elizavetadogadina@gmail.com (E.D.); kogolev@tpu.ru (D.K.); aov4@tpu.ru (A.V.); postnikov@tpu.ru (P.P.); esheremet@tpu.ru (E.S.)
2 School of Materials and Energy, University of Electronic Science and Technology of China, 610054 Chengdu, China; jinjuchen@uestc.edu.cn
3 Brazilian Nanotechnology National Laboratory (LNNano), Brazilian Center for Research in Energy and Materials (CNPEM), 13083-970 Campinas, Brazil; rafael.furlan@lnnano.cnpem.br
4 Measurement and Sensor Technology, Faculty of Electrical Engineering and Information Technology, Technische Universität Chemnitz, 09126 Chemnitz, Germany
* Correspondence: olfa.kanoun@etit.tu-chemnitz.de (O.K.); raul@tpu.ru (R.D.R.)

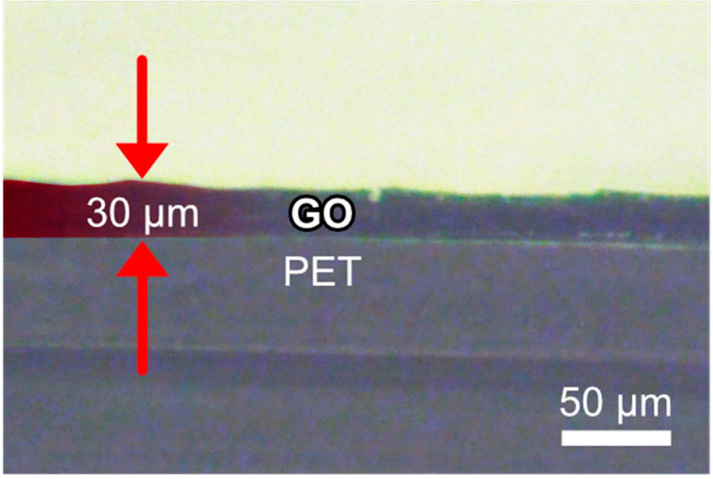

Figure S1. Cross-section optical microscopy image of the GO/PET (sample before laser treatment). The red color-marked region at the left highlights the GO film with a thickness of 30 µm. The rGO/PET composite obtained after laser processing is shown by the cross-section in Figure 1c.

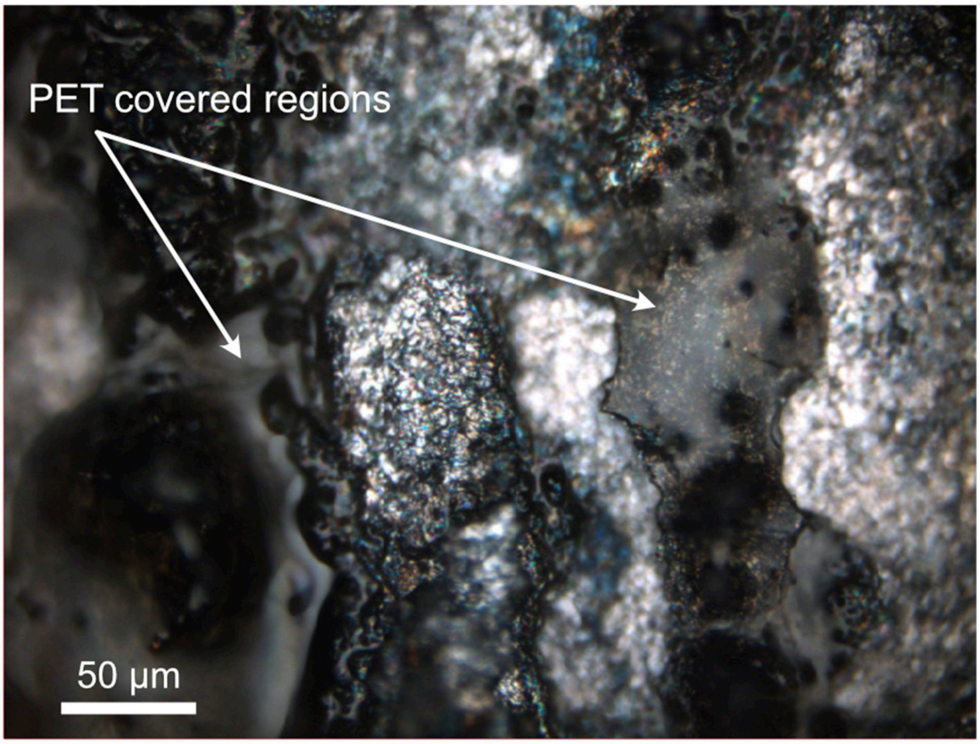

Figure S2. Optical microscopy image of pristine rGO/PET electrode.

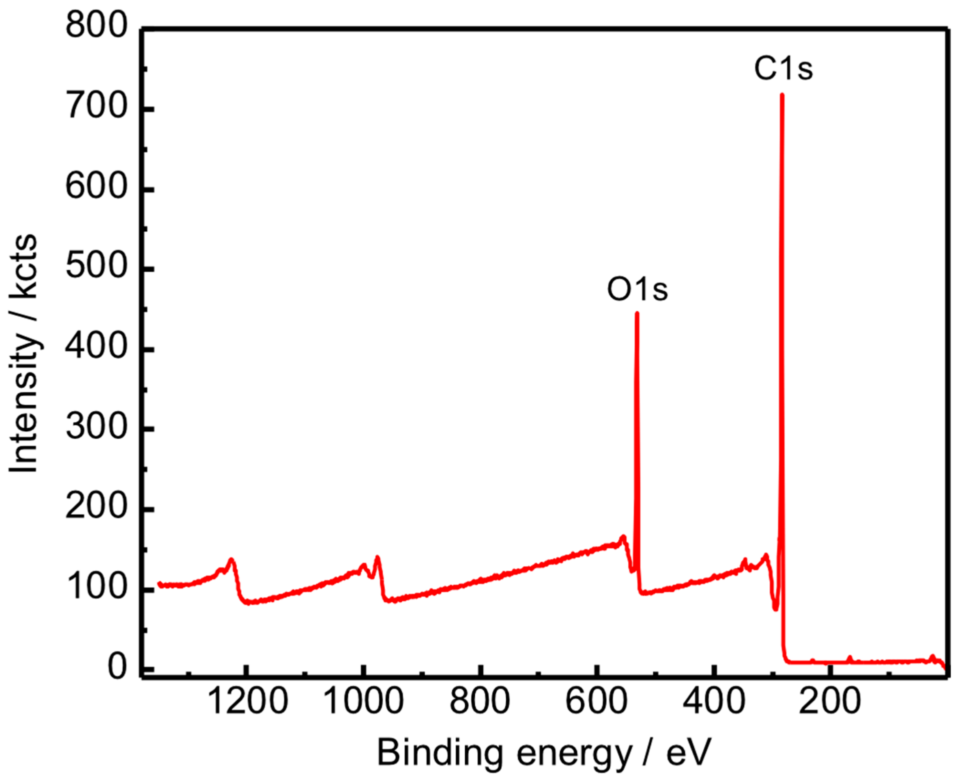

Figure S3. XPS survey spectrum from the rGO/PET pristine sample.

Table S1. Calculated values from CVs and Impedance plots after electrochemical treatment.

**Cycle №**	**$\|Z\|_{0.25 Hz}$, kΩ**	**R_{CT}, kΩ**	**C, mF cm^{-2}**
**Initial**	55.6 ± 26.5	8.9 ± 6.6	1.2 ± 0.7
**1st reduction**	16.9 ± 3.3	0.9 ± 0.4	1.5 ± 0.3
**1st oxidation**	0.6 ± 0.1	0.4 ± 0.1	12.1 ± 0.9
**2nd reduction**	0.52 ± 0.03	0.22 ± 0.02	7.2 ± 1.4
**2nd oxidation**	0.9 ± 0.1	0.44 ± 0.02	3.1 ± 1.7
**3rd reduction**	0.7 ± 0.2	0.37 ± 0.05	9.0 ± 1.9
**3rd oxidation**	5.0 ± 0.8	2.2 ± 0.2	0.9 ± 0.1
**4th reduction**	1.2 ± 0.2	0.62 ± 0.07	7.6 ± 2.8
**4th oxidation**	12.2 ± 0.6	5.1 ± 0.3	0.4 ± 0.1
**5th reduction**	1.8 ± 0.4	0.84 ± 0.02	6.7 ± 0.9
**5th oxidation**	16.9 ± 1.5	7.8 ± 0.9	0.25 ± 0.03
**6th reduction**	2.2 ± 0.5	1.1 ± 0.1	6.8 ± 0.9
**6th oxidation**	17.9 ± 1.0	9.1 ± 0.7	0.21 ± 0.02
**7th reduction**	3.5 ± 0.6	1.6 ± 0.1	5.6 ± 1.0
**7th oxidation**	18.9 ± 2.1	9.4 ± 1.0	0.25 ± 0.04
**8th reduction**	4.2 ± 0.3	2.2 ± 0.1	5.0 ± 0.5
**8th oxidation**	21.2 ± 3.6	10.8 ± 2.0	0.25 ± 0.04

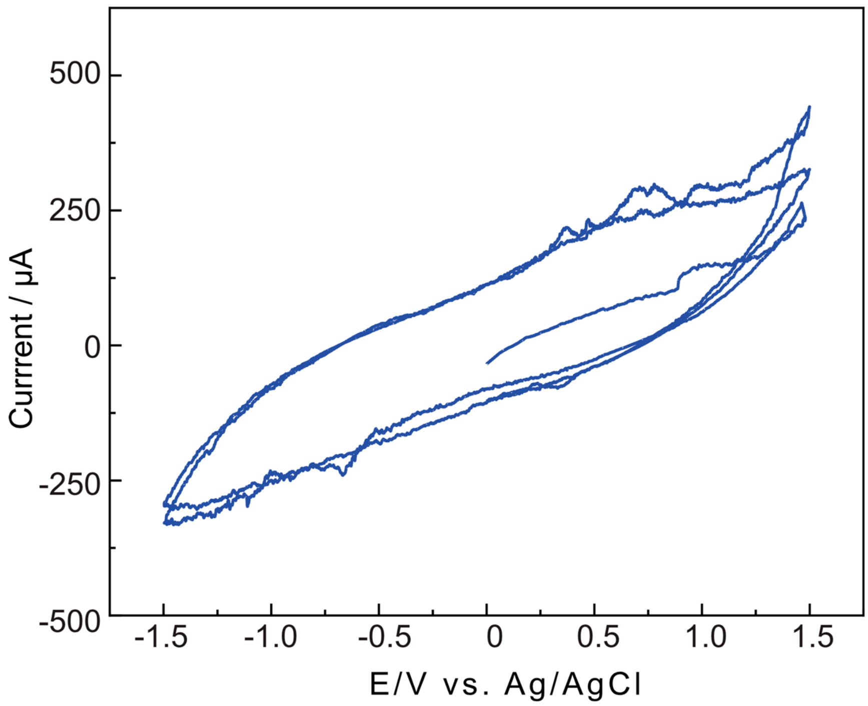

Figure S4. Typical CV after the first reduction in a following sequence 1st ox-1st red (treatment was started with the oxidation step).

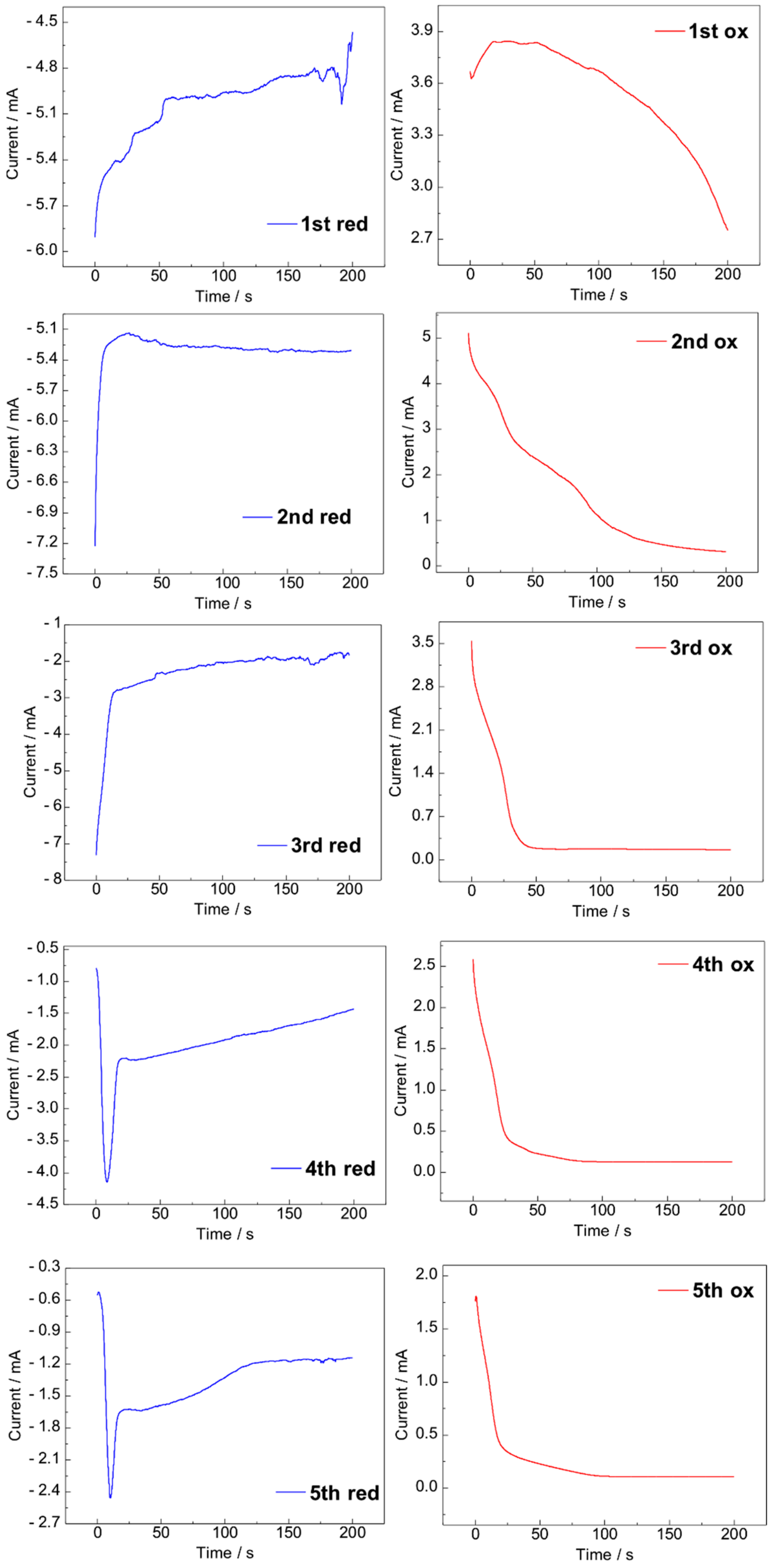

Figure S5. i-t curves from the rGO/PET electrode during each of the first 5 redox cycles.

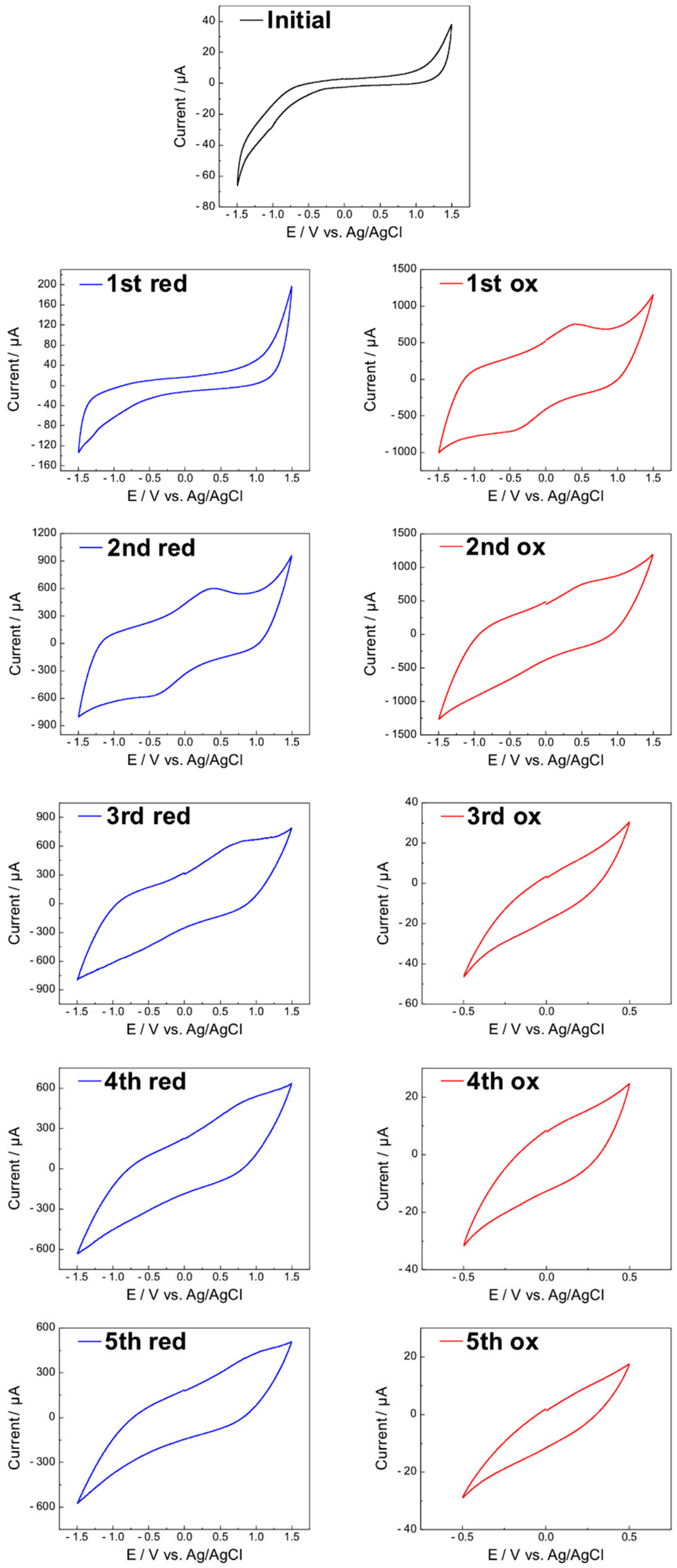

Figure S6. CVs from rGO/PET electrode after each of the first 5 redox cycles.

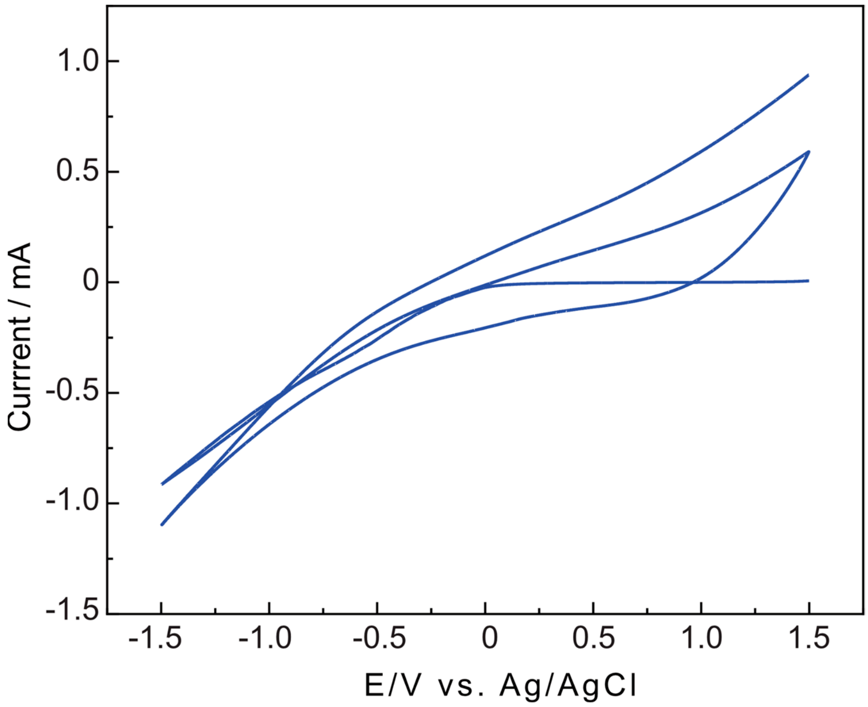

Figure S7. CV after the third oxidation cycle without changing the potential window.

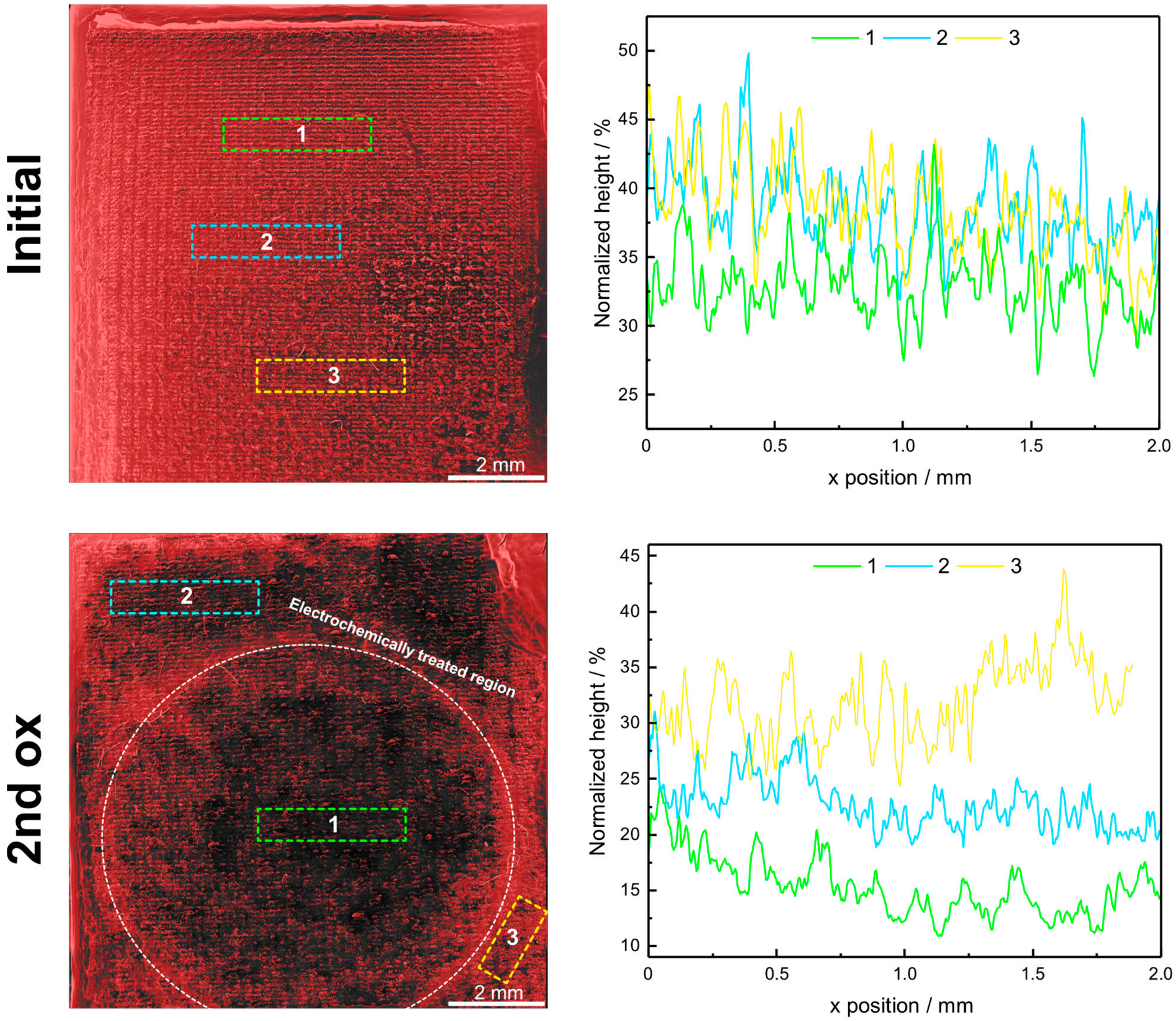

Figure S8. SEM images of the initial sample and sample after the 2nd oxidation. The red mask was created as a grain analysis to illustrate PET islands. Dashed squares show regions from which the profiles were extracted, while the white circle represents the electrochemically treated region.

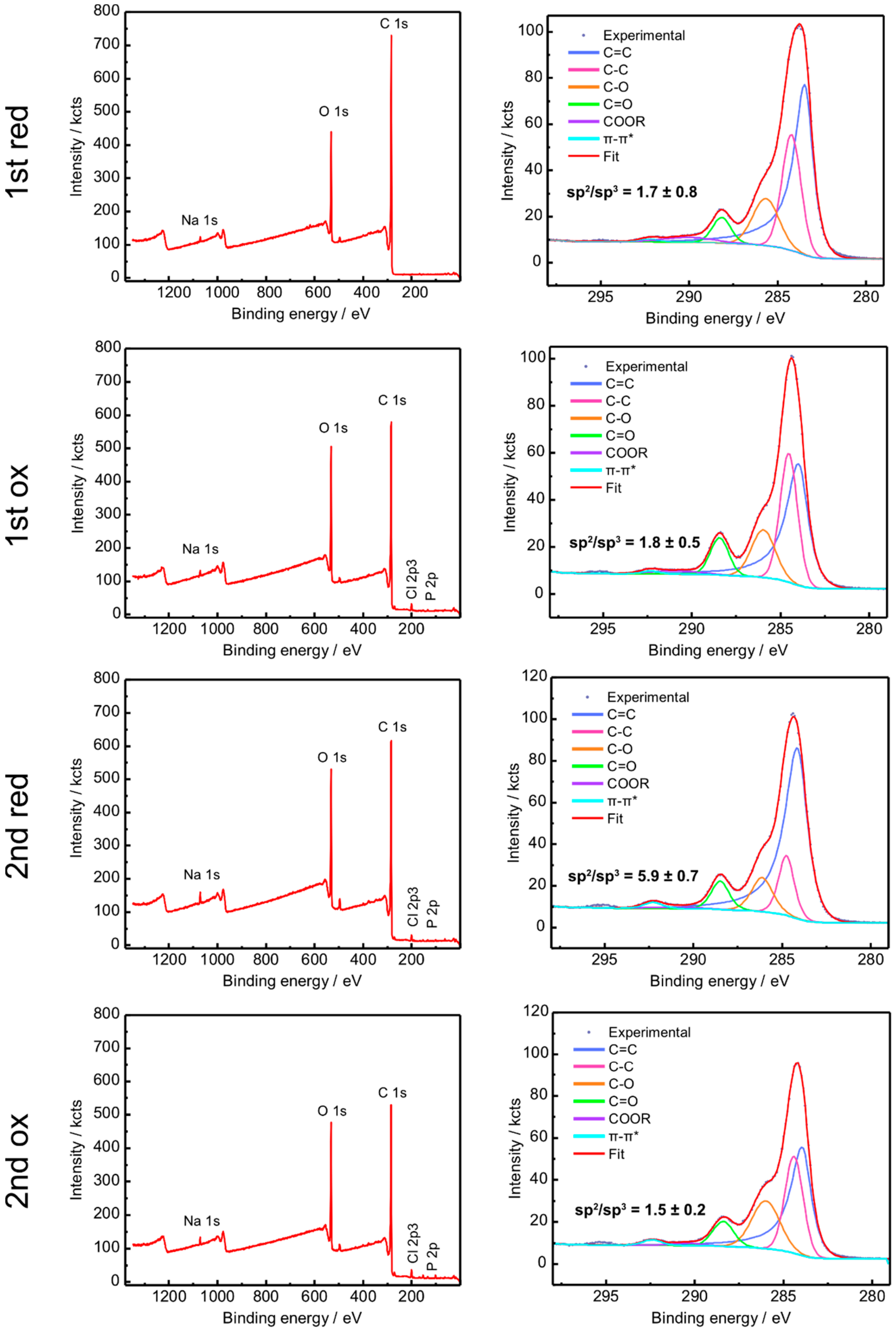

Component	Atomic %
C=C	28.8 ± 5.0
C-C	19.8 ± 8.2
C-O	7,6 ± 2.1
C=O	4.0 ± 0.5
π-π*	1.0 ± 0.6
COOR	1.2 ± 0.4

Component	Atomic %
C=C	22.6 ± 2.4
C-C	13.2 ± 2.6
C-O	10.3 ± 2.2
C=O	4.7 ± 0.1
π-π*	0.3 ± 0.1
COOR	1.2 ± 0.2

Component	Atomic %
C=C	37.5 ± 1.8
C-C	6.5 ± 0.6
C-O	4.8 ± 0.6
C=O	3.7 ± 0.2
π-π*	0.9 ± 0.1
COOR	0.3 ± 0.2

Component	Atomic %
C=C	23.0 ± 1.3
C-C	16.1 ± 2.8
C-O	8.8 ± 3.6
C=O	4.3 ± 0.2
π-π*	1.1 ± 0.2
COOR	0.6 ± 0.1

Figure S9. Survey and C1s XPS high-resolution spectra from rGO/PET electrode after two redox cycles. The tables show values of relative atomic content averaged from 3 different points per sample.

Table S2. Atomic percent of elements from XPS Survey spectra for rGO/PET samples after first two redox cycles.

Sample	Atomic %						
	**C1s**	**O1s**	**Cl2p3**	**Na1s**	**P2p**	**S2p**	**C/O ratio**
**Intital**	80.7 ± 0.3	18.03 ± 0.4	-	0.14 ± 0.05	-	0.6 ± 0.1	4.42 ± 0.11
**1st red**	83.0 ± 0.9	16.3 ± 0.8	-	0.4 ± 0.1	-	0.3 ± 0.1	5.09 ± 0.31
**1st ox**	75.0 ± 0.9	22.2 ± 0.3	1.0 ± 0.1	0.6 ± 0.1	0.7 ± 0.1	0.3 ± 0.1	3.38 ± 0.07
**2nd red**	75.4 ± 0.8	21.3 ± 0.9	0.8 ± 0.1	0.8 ± 0.1	0.4 ± 0.1	-	3.54 ± 0.20
**2nd ox**	74.4 ± 0.9	20.9 ± 0.1	2.5 ± 0.3	0.6 ± 0.1	0.7 ± 0.1	0.4 ± 0.2	3.57 ± 0.05

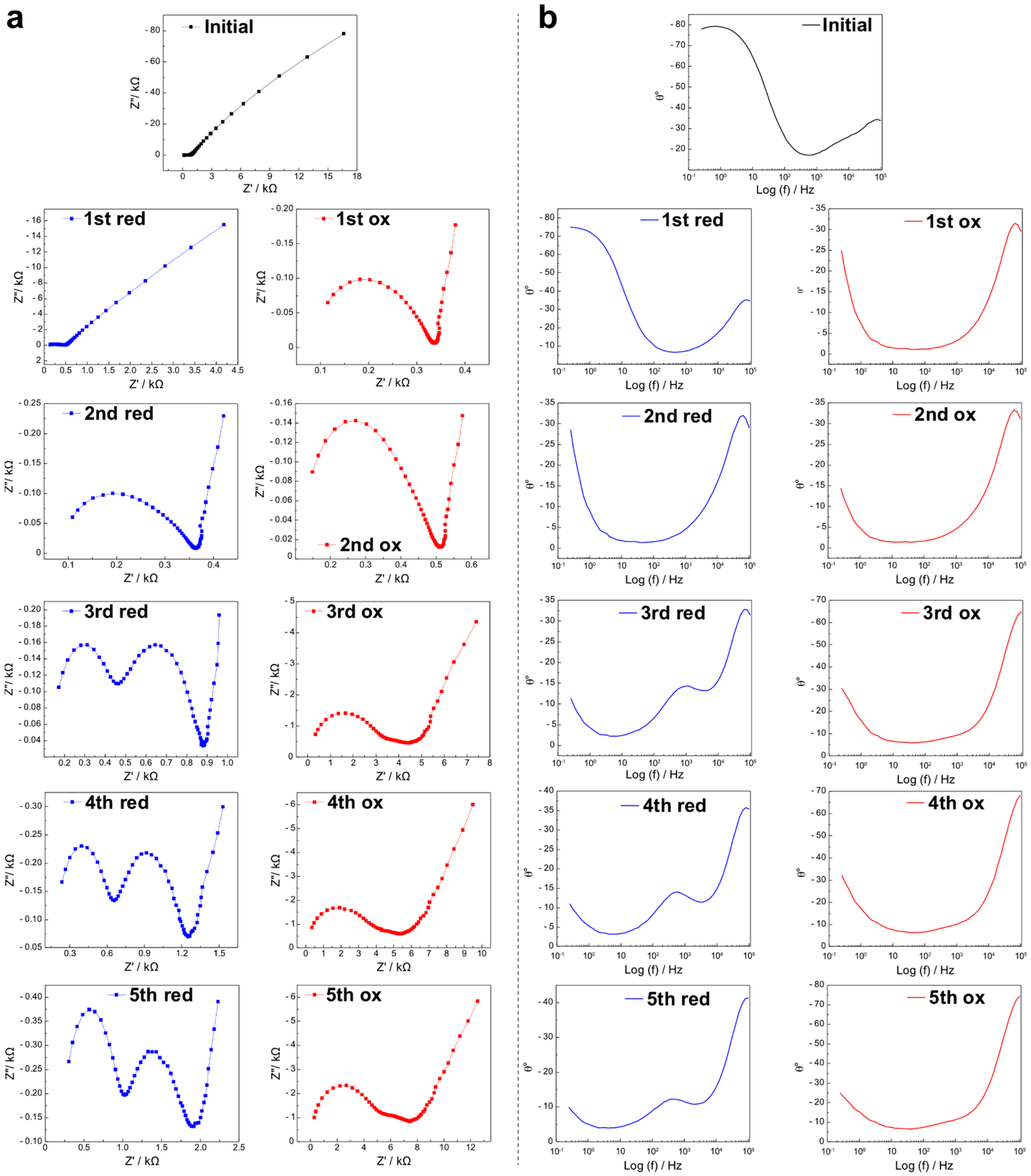

Figure S10. (**a**) Nyquist plots and (**b**) phase angle plots from the rGO/PET electrode after each of the first 5 redox cycles.

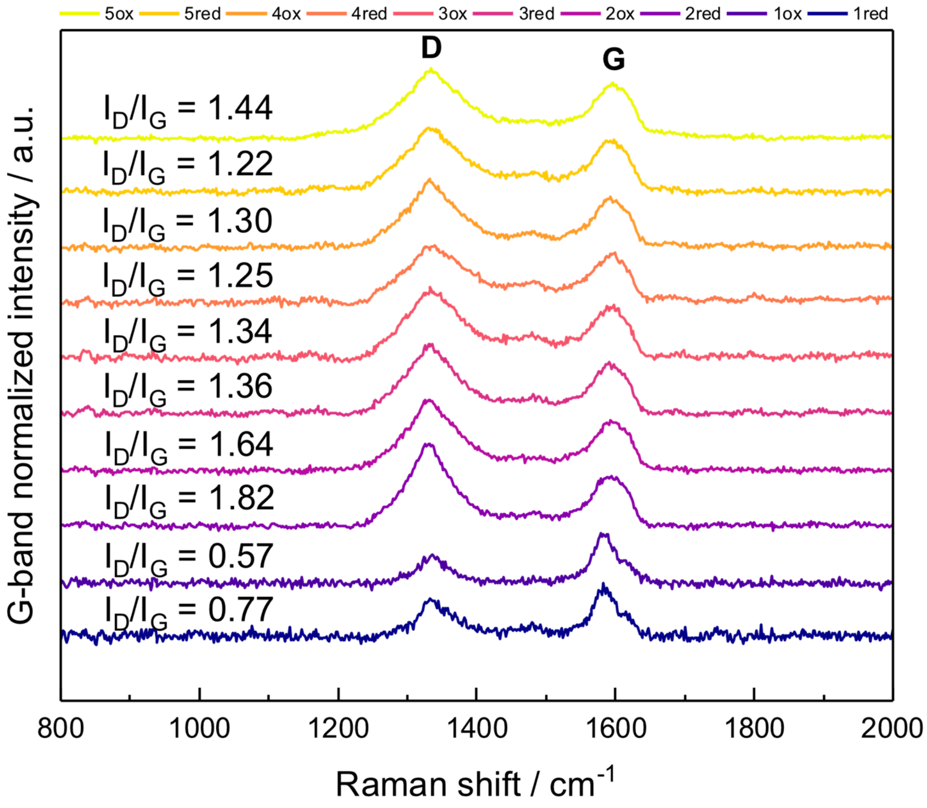

Figure S11. Raman spectra from the rGO/PET electrodes after each of 5 electrochemically treatment cycles.

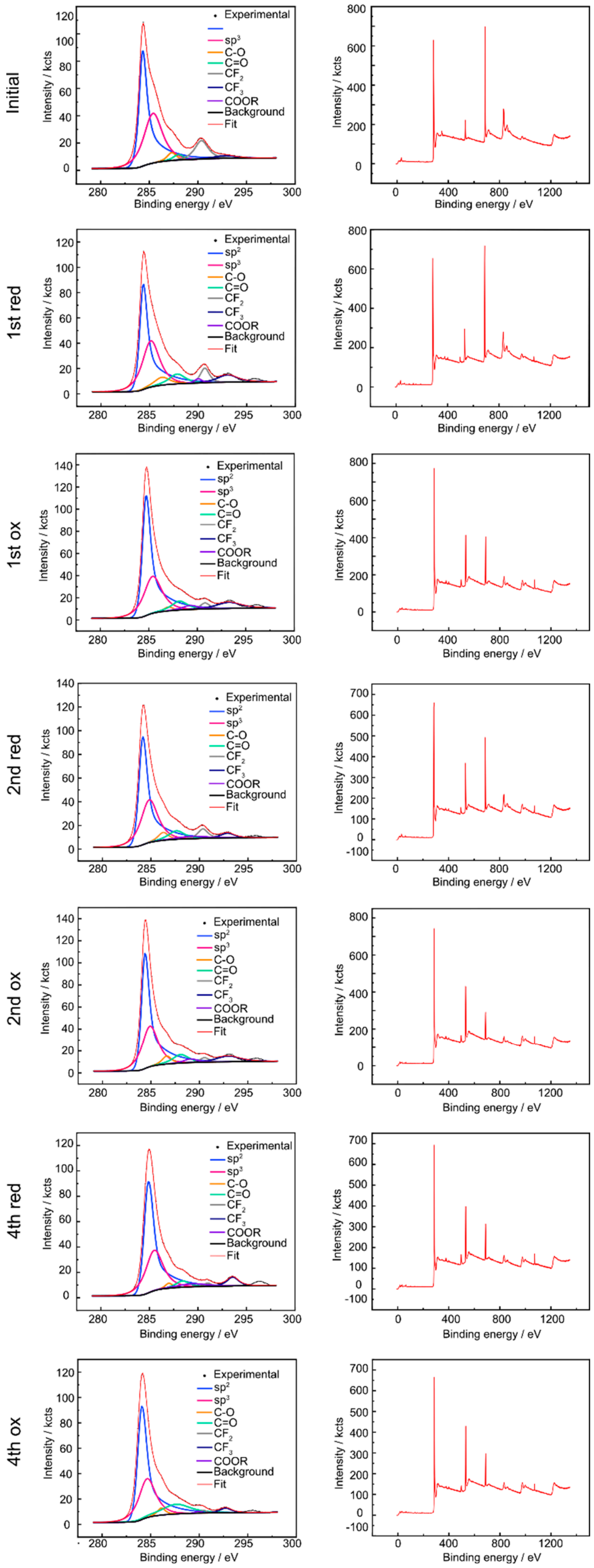

Figure S12. Survey and C1s XPS high-resolution spectra from rGO/PVDF electrode after several redox cycles.

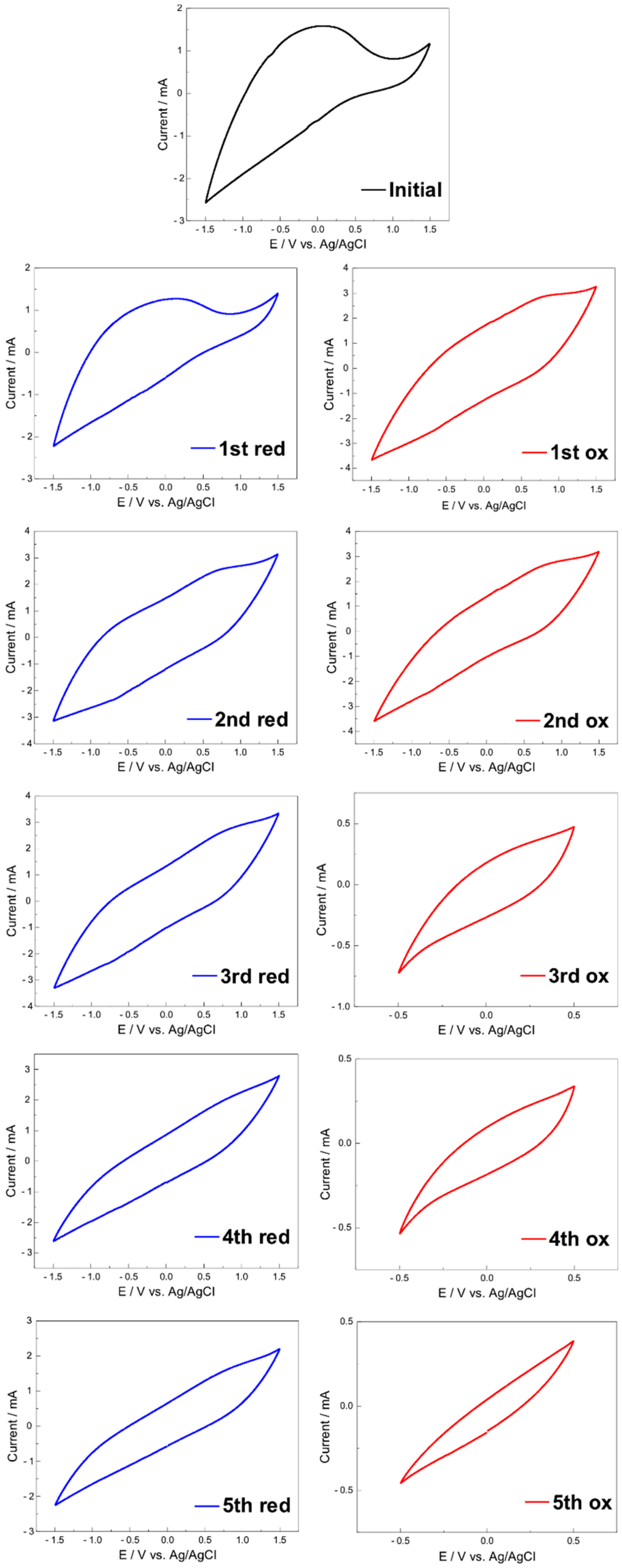

Figure S13. CVs from rGO/PVDF electrode after each of the first 5 redox cycles.

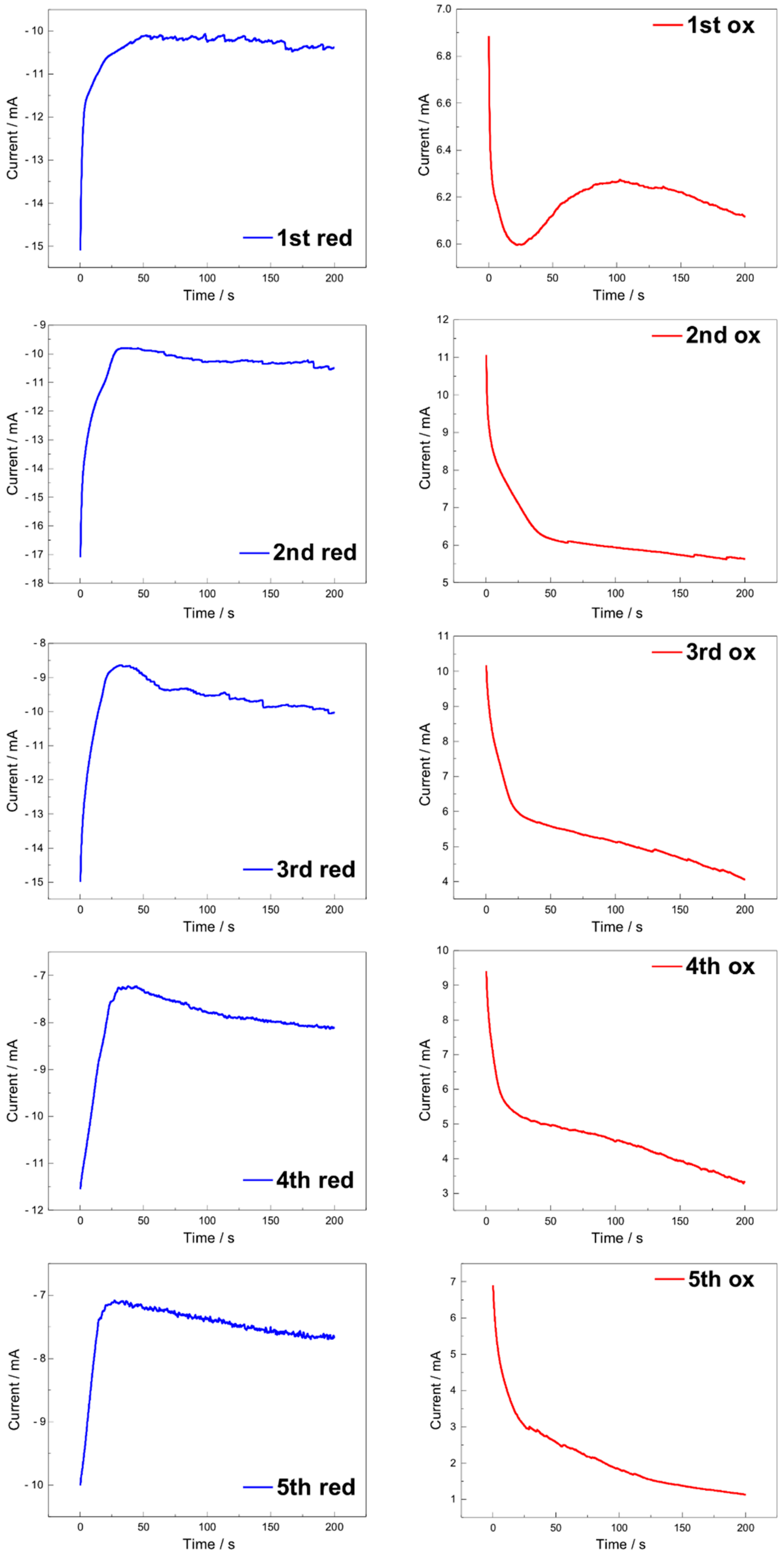

Figure S14. i-t curves from rGO/PVDF electrode after each of the first 5 redox cycles.

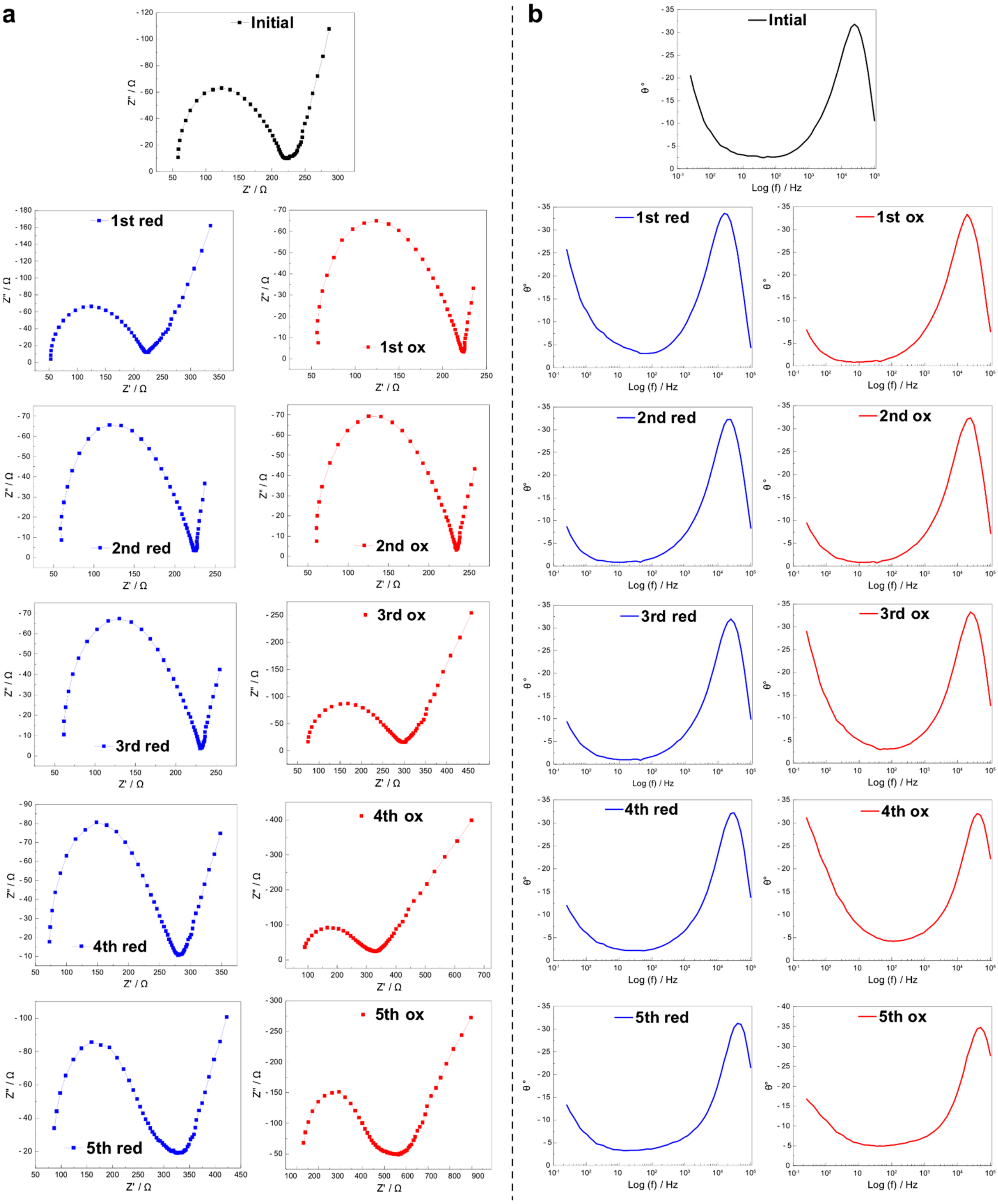

Figure S15. (a) Nyquist plots and (b) phase angle plots from the rGO/PVDF electrode after each of the first 5 redox cycles.

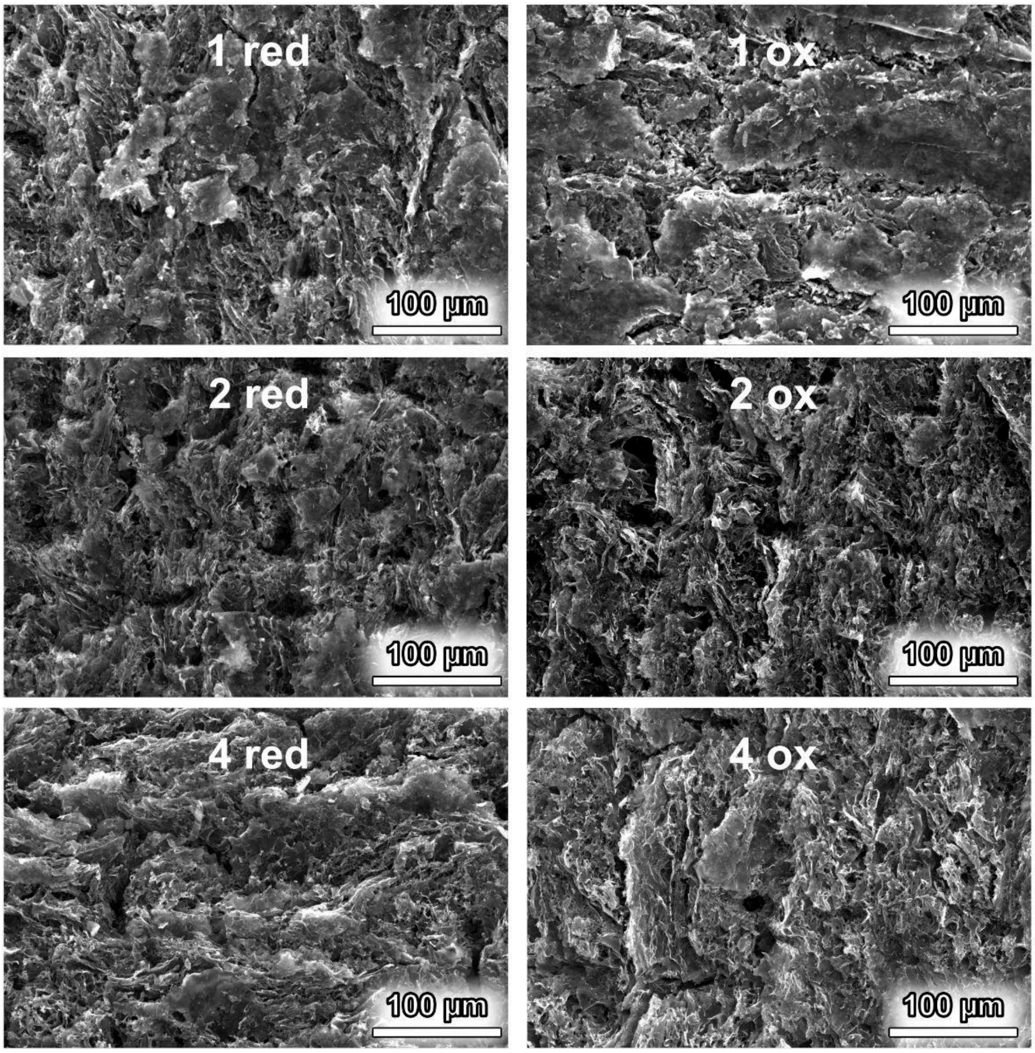

Figure S16. SEM of rGO/PET electrode after several redox cycles.

Table S3. C/O ratio extracted from XPS survey spectra from different treatment cycles of rGO/PVDF.

Sample	C/O ratio
Initial	15.2 ± 0.5
1st reduction	9.0 ± 0.1
1st oxidation	5.7 ± 0.3
2nd reduction	6.5 ± 0.3
2nd oxidation	5.0 ± 0.4
4th reduction	5.5 ± 0.1
4th oxidation	5.1 ± 0.1

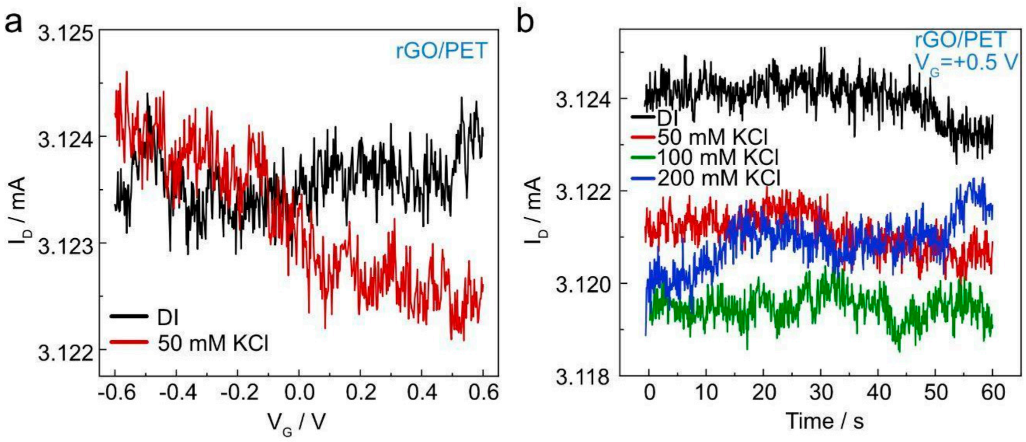

Figure S17. (**a**) Transfer characteristic of pristine rGO/PET in DI and 50 mM KCl. (**b**) Respective current-time at V_G = +0.5 V and V_{DS} = -0.5 V for varied KCl concentrations.

Note S1.

Charge carriers' mobility for all the samples was calculated according the formula (1):

$$\mu = \frac{\Delta I_D}{\Delta V_G} \cdot \frac{L}{W \cdot V_{DS} \cdot C} \quad (1);$$

where μ - charge carriers' mobility, $\frac{\Delta I_D}{\Delta V_G}$ - slope of the linear region of transfer characteristic, L, W - the length and the width of a channel, V_{DS} - source-drain voltage, C - cpecific capacitance.
